# Viral Decay Kinetics in the Highly Active Antiretroviral Therapy-Treated Rhesus Macaque Model of AIDS

**DOI:** 10.1371/journal.pone.0011640

**Published:** 2010-07-23

**Authors:** Jesse D. Deere, Joanne Higgins, Elda Cannavo, Andradi Villalobos, Lourdes Adamson, Emilie Fromentin, Raymond F. Schinazi, Paul A. Luciw, Thomas W. North

**Affiliations:** 1 Center for Comparative Medicine, University of California Davis, Davis, California, United States of America; 2 Department of Pathology, School of Medicine, University of California Davis, Davis, California, United States of America; 3 Department of Veterinary Molecular Biosciences, University of California Davis, Davis, California, United States of America; 4 Center for AIDS Research, Laboratory of Biochemical Pharmacology, Department of Pediatrics, Emory University School of Medicine, Veterans Affairs Medical Center, Decatur, Georgia, United States of America; University of Pittsburgh, United States of America

## Abstract

To prevent progression to AIDS, persons infected with human immunodeficiency virus type 1 (HIV-1) must remain on highly active antiretroviral therapy (HAART) indefinitely since this modality does not eradicate the virus. The mechanisms involved in viral persistence during HAART are poorly understood, but an animal model of HAART could help elucidate these mechanisms and enable studies of HIV-1 eradication strategies. Due to the specificity of non-nucleoside reverse transcriptase (RT) inhibitors (NNRTIs) for HIV-1, we have used RT-SHIV, a chimeric virus of simian immunodeficiency virus with RT from HIV-1. This virus is susceptible to NNRTIs and causes an AIDS-like disease in rhesus macaques. In this study, two groups of HAART-treated, RT-SHIV-infected macaques were analyzed to determine viral decay kinetics. In the first group, viral loads were monitored with a standard TaqMan RT-PCR assay with a limit of detection of 50 viral RNA copies per mL. Upon initiation of HAART, viremia decayed in a bi-phasic manner with half-lives of 1.7 and 8.5 days, respectively. A third phase was observed with little further decay. In the second group, the macaques were followed longitudinally with a more sensitive assay utilizing ultracentrifugation to concentrate virus from plasma. Bi-phasic decay of viral RNA was also observed in these animals with half-lives of 1.8 and 5.8 days. Viral loads in these animals during a third phase ranged from 2–58 RNA copies/mL, with little decay over time. The viral decay kinetics observed in these macaques are similar to those reported for HIV-1 infected humans. These results demonstrate that low-level viremia persists in RT-SHIV-infected macaques despite a HAART regimen commonly used in humans.

## Introduction

Highly active antiretroviral therapy (HAART) is a combination of drugs, usually three or more from two or more classes, which serves as a means to long-term control of replication of the lentivirus, human immunodeficiency virus type 1 (HIV-1) [1], [2], [3]. Effective HAART can reduce viremia to below the detectable limits of conventional clinical assays in many persons able to adhere to the treatment regimen, drastically reducing their progression to acquired immune deficiency syndrome (AIDS) and extending life. However, the development of more sensitive assays has demonstrated that continued low-level viremia persists in most subjects despite many years of viral suppression by HAART [4], [5], [6], [7]. Additionally, viremia rebounds when treatment stops [8], [9]. HIV-1 is not eradicated with current drug regimens, and thus infected persons must remain on HAART indefinitely.

Several hypotheses have been proposed recently to explain the persistence of HIV-1 despite suppressive HAART [10], [11]. These hypotheses are not mutually exclusive, and the mechanisms of persistence might vary between individuals [12], [13]. Because current HAART is only capable of blocking new rounds of infection, this treatment modality is unable to eliminate cells containing an integrated viral genome. Upon initiation of HAART, viremia decays in a bi-phasic manner to low levels [4], [14], [15], [16], [17]. The phases of decay of viremia represent both the turnover of virions in plasma as well as the turnover of infected cells. A recent publication has identified a slow, third phase of decay followed by a fourth phase with no apparent further decay [6]. Infected resting memory CD4^+^ T cells and macrophages are examples of stable reservoirs that occur early in infection and persist for years despite suppressive HAART [18], [19], [20], [21], [22], [23]. The generally low cellular activation state of these cells could prevent the virus from completing its replication cycle. Occasional immune activation of these cells might allow for complete viral transcription and lead to assembly and release of virus, accounting for the observed low-level viremia. There may be additional stable, long-lived cells that are infected and release virus continuously [5], [22]. Also, tissues or cell types with restricted drug access might exist within a person, allowing for low-level residual replication [12], [13], [24], [25]. The observed residual viremia might continue to reseed reservoirs thereby prohibiting their decay.

Attempts to address mechanisms of viral persistence have been limited partly because extensive tissue samples during suppressive HAART are not available for analysis. Even if possible, HIV-1 eradication will not be proven until infected individuals can be removed from HAART without viral rebound [26]. However, ethical concerns surround structured treatment interruptions [8], [27]. A well developed animal model for HAART will enable extensive tissue analysis to identify the source of residual viremia. This approach could lead to a better understanding of viral persistence during suppressive therapy. An appropriate animal model would also lend itself to evaluation of higher risk treatment regimens that are not feasible in human studies, including the use of viral rebound as an endpoint.

Another lentivirus, simian immunodeficiency virus (SIV), causes AIDS in macaques and is sensitive to many of the approved nucleoside analog reverse transcriptase (RT) inhibitors (NRTIs) and protease inhibitors (PIs) used in current HAART regimens [28], [29], [30]. SIV has been used as a model of HIV-1 to study pathogenesis, immune responses, vaccines, and therapy [29], [31], [32]. Recently, SIV was used to study viral reservoirs in pig-tailed macaques during treatment with a HAART regimen [33].

A major limitation of using SIV infection of macaques to model HAART is that the non-nucleoside reverse transcriptase inhibitors (NNRTIs) are not effective against the SIV RT. The NNRTI efavirenz is a component of a combination currently recommended as an initial HAART regimen [34]. Accordingly, NNRTIs should be an option in an animal model attempting to address viral persistence during HAART.

Non-human primate models that can utilize NNRTIs have been developed [35], [36], [37], [38]. One of the rhesus macaque models uses virus consisting of the backbone of the pathogenic molecular clone SIVmac239 with the HIV-1 RT from clone HXBc2 (RT-SHIV) [38]. RT-SHIV, which causes simian AIDS in rhesus macaques, is sensitive to several NRTIs, PIs, and NNRTIs [28], [38], [39], [40], [41]. We recently demonstrated that RT-SHIV-infected rhesus macaques treated with an efavirenz-based HAART regimen (which included lamivudine and tenofovir) models treatment of HIV-1 infection in humans [37]. Plasma virus loads (VLs) in treated animals drop to below the level of detection (50 copies of viral RNA per mL) during HAART, and VLs rebound when treatment is terminated [37].

We hypothesized that low-level viremia persists in RT-SHIV-infected rhesus macaques despite suppressive HAART, and that this viremia could be detected using a more sensitive assay. Herein, we report for the first time the VL kinetics of rhesus macaques infected with RT-SHIV and treated with HAART. VLs were analyzed both at necropsy and longitudinally. The results demonstrated that despite suppressive HAART, detectable low-level viremia persisted in the animals. These data also suggest that the RT-SHIV/rhesus macaque model will enable critical studies of mechanisms of viral persistence during HAART.

## Materials and Methods

### Ethics Statement

All animals were from the retrovirus-free colony of the California National Primate Research Center (CNPRC), which operates according to the Guide for the Care and Use of Laboratory Animals prepared by the Committee on Care and Use of Laboratory Animals of the Institute of Laboratory Animal Resources, National Research Council. The studies were approved by University of California, Davis Institutional Animal Care and Use Committee (IACUC). This institution is accredited by the Association for Assessment and Accreditation of Laboratory Animal Care, International (AAALAC). This institution has an Animal Welfare Assurance on file with the Office of Laboratory Animal Welfare (OLAW). All possible efforts were made to minimize animal pain and discomfort. Analgesics were administered at the discretion of the CNPRC veterinary staff. When necessary, animals were immobilized with ketamine-HCl (Parke-Davis, Morris Plains, NJ, USA), 10 mg/kg body weight, injected intramuscularly. At necropsy, macaques were sedated with ketamine-HCl and then humanely euthanized with a barbiturate overdose and peripheral blood was collected.

### Virus and cells

RT-SHIV stocks were prepared by transfecting CEMx174 cells as described previously [28], [41]. The 5′-half clone encoding the RT of HIV-1 clone HXBc2 [38] was provided by J. Sodroski, Harvard Medical School, Boston, Mass. The 3′-half clone encodes a full-length nef open reading frame as described [42]. CEMx174 cells were grown as previously described [37]. Virus stocks were prepared as previously described [37] and had the T-to-C substitution at position 8 of the SIV tRNA primer binding site, which is necessary for rapid replication of RT-SHIV [43].

Feline leukemia virus (FeLV) was used to aid in pelleting of RT-SHIV during the ultracentrifugation virus load assay (UVLA) as described below. Stocks of FeLV were collected from the media supernatant of the chronically infected feline lymphoblastoid cell line FL74 [44]. Cell culture supernatants containing FeLV were frozen in aliquots at −80°C.

### Preparation and administration of drug

Two groups of 12 juvenile rhesus macaques (*Macaca mulatta*) weighing approximately 2 to 4 kg were infected intravenously with approximately 10^5^ 50% tissue culture infectious doses of RT-SHIV grown in CEMx174 cells. Six weeks post-infection, nine of the animals in each group began a HAART regimen consisting of tenofovir (PMPA), emtricitabine (FTC), and efavirenz (EFV; Sustiva). The remaining three animals in each group served as untreated control animals. FTC and PMPA were provided by Gilead Sciences (Foster City, CA, USA). EFV was provided by Bristol-Myers Squibb (Wallingford, CT, USA) for the first group of animals, and was purchased from a pharmacy for the second group. EFV was fed at 200 mg per day by mixing the contents of a 200 mg Sustiva capsule into food such as peanut butter sandwiches. Stock solutions of FTC were prepared in phosphate buffered saline (pH 7.4). PMPA was suspended in distilled water with NaOH added to a final pH of 7.0. FTC and PMPA stocks were filter-sterilized and stored at 4°C. These NRTIs were administered subcutaneously at a regimen of 16 mg per kg body weight once daily for FTC and 30 mg per kg body weight once daily for PMPA. Drug dosages were adjusted weekly according to body weight. The dose of PMPA was reduced to 15 mg/kg per day after 15 weeks of treatment to reduce the risk of renal toxicity [45].

### Sample collection

EDTA-anticoagulated blood samples were taken regularly and plasma was stored at −80°C until RNA extraction and quantification. In the group of animals analyzed at necropsy, 1 mL samples were drawn weekly for the first 10 weeks followed by bi-monthly with occasional weekly sampling until necropsy, when larger volumes were collected. In the animals analyzed longitudinally, 1 mL samples were taken weekly for 10 weeks post-infection, then larger samples were taken every two weeks through week 16, and then every four weeks through week 34.

### Isolation of RNA from plasma

Plasma samples archived at −80°C were analyzed for viral loads using a method similar to previously published assays for HIV-1 and SIV [7], [46]. In preliminary experiments, plasma was thawed from −80°C, followed by ultracentrifugation to concentrate the virus, and then RNA was quantified by TaqMan RT-PCR as described below. In many of the samples a flocculent, waxy substance was present in the tubes after RNA isolation. The substance was not soluble in water and interfered with the TaqMan RT-PCR step. This problem was resolved by including an initial, low-speed centrifugation of the recently thawed plasma at 1,100×*g* for 10 min. After this first low-speed centrifugation, plasma supernatants were transferred to fresh 15 mL tubes. For the standard virus load assay (SVLA), viral RNA was isolated from 140 µL of the clarified plasma using the Qiagen viral RNA kit (Valencia, CA, USA) according to the manufacturer's protocol. RNA was eluted from the columns with 40 µL of molecular grade water. The remaining plasma sample was analyzed using the UVLA by diluting the plasma (ranging from 1.9 to 6.0 mL) with tris-buffered saline pH 7.0 (TBS) to a final volume of 6 mL. To aid in pelleting during ultracentrifugation, FeLV stock was added to each sample at 1×10^6^ RNA copies in 500 µL of TBS. Each sample was transferred to a 13.2 mL Beckman polyallomer ultracentrifuge tube (Brea, CA, USA) and centrifuged in a Beckman Optima LE-80K ultracentrifuge at 170,000×g for 35 min at 4°C in a Beckman SW41 Ti rotor. After ultracentrifugation, the supernatant was removed and the pelleted virions were re-suspended in 100 µL of 5 mM Tris-HCl (pH 8.0) containing 200 µg of Proteinase K (Sigma Aldrich, St. Louis, MO, USA) followed by a 30 min incubation at 55°C. Virions were lysed by the addition of 400 µL of 5.8 M guanidinium isothiocyanate (Sigma Aldrich, St. Louis, MO, USA) containing 200 µg of glycogen (Sigma Aldrich) and each sample was transferred to a 1.5 mL tube. The samples were then pulse-vortexed for 15 sec followed by incubation for 10 min at room temperature. RNA was precipitated by adding 500 µL of 100% isopropanol followed by inverting the tubes several times and then 15 sec pulse vortexing. These samples were incubated on ice for 40–60 min before centrifugation at 21,000×g for 25 min at 4°C to pellet RNA. The supernatant was removed and the pellet was washed with 900 µL of 70% ethanol followed by centrifugation at 21,000×g for 15 min at 4°C. The supernatant was removed and discarded and the RNA was allowed to air dry for several minutes before re-suspension in 55 µL of molecular grade water. All reagents were molecular grade and certified RNase/DNase free.

### TaqMan RT-PCR

RT-SHIV RNA was quantified using TaqMan RT-PCR as previously described [47]. Control reactions in the absence of RT were included for each sample. Occasional samples had a detectable level of DNA. In these samples, viral DNA copies per mL were calculated and subtracted from the reactions containing reverse transcriptase to determine viral RNA copies per mL. In preliminary experiments, DNase treatments interfered with subsequent TaqMan reactions. As a result, they were not performed on the reported samples.

FeLV RNA was also quantified in the samples after ultracentrifugation in order to assess recovery of virus. Duplicate 25 µL TaqMan RT-PCR reactions containing 5 µL of RNA sample were performed using the primer-probe set targeting the unique region (U3) of the FeLV long terminal repeat described by Tandon, et al. [48] with the same reaction conditions used for RT-SHIV quantification.

### Efavirenz quantification

After methanol extraction from plasma, EFV was separated by high-performance liquid chromatography on a Hypersil-GOLD-C18 column using a gradient of water and acetonitrile and detected by electrospray ionisation/tandem mass spectrometry in the negative mode (m/z 314> m/z 69).

### Statistical analysis

Statistical analyses were performed using GraphPad Prism version 5.01 for Windows, GraphPad Software (San Diego, CA, USA) www.graphpad.com. The viral load decay analyses were performed using Joinpoint Regression Program Version 3.4.1. September 2009; Statistical Research and Applications Branch, National Cancer Institute (Bethesda, MD, USA). Graphs were reproduced in Microsoft Office Excel 2003 for formatting standards.

## Results

### Analysis of viremia at necropsy

**Figure 1:**
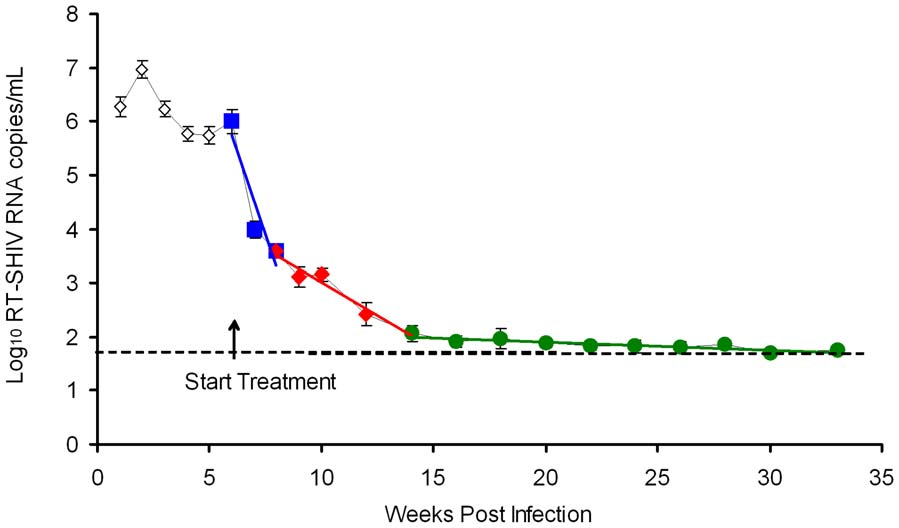
RT-SHIV viral decay kinetics of HAART-treated macaques. The average plasma virus load of 9 RT-SHIV-infected, HAART-treated macaques using the standard virus load assay (SVLA). Colored lines indicate linear regression analysis. Error bars indicate standard error of the mean. The dashed line indicates the limit of detection of the SVLA (50 RNA copies/mL).

In the first study, VLs were monitored throughout treatment using the SVLA. Upon initiation of HAART, the average VL decayed to the limit of detection of the SVLA (Fig. 1). The value of 50 RNA copies/mL was used to calculate the average when RNA was not detected using the SVLA. All nine treated animals eventually achieved a VL below the limit of detection of the SVLA, with occasional positive samples. Linear regression analysis of the average VL over the duration of HAART was performed (Fig.1). The best fit model divided the average VL upon initiation of HAART (6 through 33 weeks post-infection) into three distinct phases represented by lines with slopes of −0.40 (95% Confidence Interval, CI, −0.30 to −0.50), −0.082 (95% CI −0.06 to −0.11), and −0.004 (95% CI −0.0009 to −0.009; p = 0.02). Due to the exponential nature of the viral decay, as described by Ho et al. for HIV-1 [14], the viral half-lives based upon these slopes were estimated to be 1.7 (95% CI 1.4 to 2.3), 8.5 (95% CI 6.3 to 11.6), and 170 (95% CI 77 to 170) days, respectively (Table 1). These data demonstrate that upon initiation of HAART, viremia decays in a bi-phasic manner to the limit of detection of the SVLA. However, the third phase of viral decay is only an estimate because most of the samples during the third phase were below the limit of detection of the SVLA and were assigned the value of 50 RNA copies/mL for the purpose of calculations.

**Table 1:** Comparison of decay of viral RNA in plasma^a^.

Phase	RT-SHIV		SIV	HIV-1		
1	1.7 (1.4 to 2.3)^b^	1.8 (1.6 to 2.3)^c^	1.33^d^	2.1^e^	1.22^f^	1.5^g^
2	8.5 (6.3 to 11.6)^b^	5.8 (5.0 to 6.9)^c^	18.5^d^		24.9^f^	28^g^
3						273^g^
4						Infinite^g^

a Comparison of viral RNA half-lives in days from the indicated phases of RT-SHIV, SIV, or HIV-1 decay during HAART.

b Group one macaques with 95% CI.

c Group two macaques with 95% CI.

d Dinoso et al. [33].

e Ho et al. [14].

f Havlir et al. [4].

g Palmer et al. [6].

The animals continued on HAART through week 33 post-infection when five of the animals were necropsied and plasma was collected (macaques: 35339, 35342, 35343, 35349, and 35389). HAART was stopped in the remaining 4 of the treated animals to allow for viral rebound and subsequent necropsy (macaques: 35685, 35913, 35940, and 36098).

**Figure 2:**
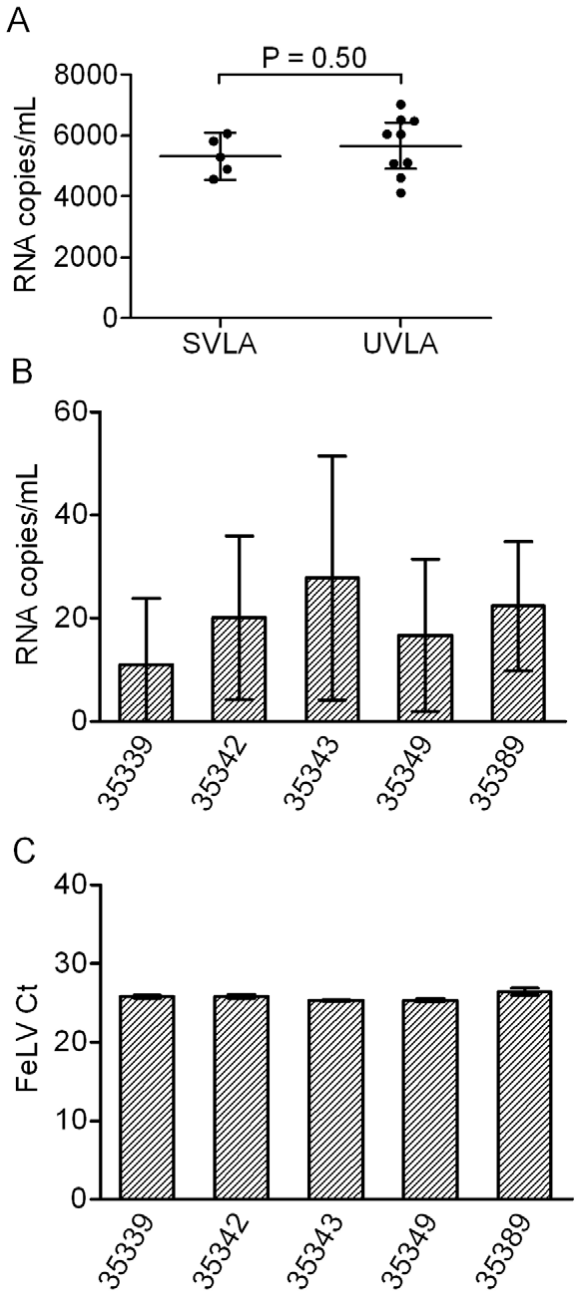
Establishment of the UVLA and analysis of plasma virus loads in macaques at necropsy. (A) Comparison of RT-SHIV RNA loads determined using the standard virus load assay (SVLA) or the ultracentrifugation virus load assay (UVLA) from macaque 35940, a macaque that was taken off HAART to allow for viral rebound. Triplicate assays with triplicate TaqMan RT-PCR for UVLA, single TaqMan RT-PCR for the SVLA. Mean with 95% confidence intervals and the results of an unpaired t test. (B) Plasma RT-SHIV RNA analysis at necropsy of 5 HAART-treated macaques. Assays were done in duplicate with triplicate TaqMan RT-PCR except macaque 35342 which was sampled in triplicate with triplicate TaqMan RT-PCR. Mean with 95% confidence intervals. (C) FeLV RNA analysis of the samples displayed in Figure 2B. Cycle threshold (Ct) obtained from TaqMan RT-PCR for FeLV analyzed in duplicate. Mean with SD.

To determine the level of residual viremia in HAART-treated, RT-SHIV-infected macaques, the HIV-1 single copy assay [7] was adapted for detection of RT-SHIV RNA. The reproducibility of this adapted assay was determined by measuring RNA in three separate aliquots of archived plasma and comparing the results with those obtained using the SVLA. These samples were taken from plasma collected at necropsy from macaque 35940 on week 47 post-infection, 14 weeks after stopping HAART (Fig. 2A). This animal had a moderate VL with an average determined using the SVLA and the UVLA of 5,300 and 5,700 RNA copies per mL, respectively (Fig. 2A). These values were within the 95% CI, and an unpaired two-tailed t test determined no statistical difference between the mean VLs measured by the two assays (Fig. 2A). These data demonstrate that the UVLA is reproducible and that the two virus load assays are comparable in the detection of RT-SHIV viremia in rhesus macaques that have moderate VLs.

VLs from the five HAART-treated monkeys that were necropsied during treatment were analyzed using both assays (Fig. 2B). None of these macaques had a VL detectable using the SVLA, but RNA was detected in all five using the UVLA (Fig. 2B). The average VLs at necropsy ranged from 11–28 RNA copies per mL (Fig. 2B). A one-way ANOVA indicated no significant difference between the average VLs (*p* = 0.54). Occasionally, a sample was undetectable even with the UVLA. Not all samples contained the same plasma volume, so the UVLA limit of detection of each sample was determined based upon the volume of plasma and the previously published line equation for TaqMan RT-PCR [47]. The average VLs were calculated by including the limit of detection values for the TaqMan RT-PCR replicates that were not detected using the UVLA. As a result, Figure 2B might slightly over-estimate the average VLs. However, these data demonstrate detectable viremia at necropsy despite 26 weeks of effective HAART in RT-SHIV-infected rhesus macaques.

To aid in pelleting of RT-SHIV from plasma during the ultracentrifugation step, 10^6^ RNA copies of FeLV stock were added to each plasma sample. Subsequently, FeLV RNA was quantified in the isolated RNA sample in order to monitor for recovery of viral RNA, serving as an internal standard (Fig. 2C). The average FeLV cycle threshold (C_t_) for these samples was 25.8 with a standard deviation of 0.64 C_t_. All of the samples were within 2 standard deviations of this mean FeLV C_t_ except for 1 plasma sample from macaque 35389 which was high, indicating a poor recovery of virus (average FeLV C_t_ of 33.4); this sample was excluded from analysis.

### Longitudinal analysis of viremia

Based on the observation that low-level viremia is detectable at necropsy in RT-SHIV-infected, HAART-treated macaques, a second study was conducted to analyze viremia longitudinally in macaques. This experiment utilized more frequent sampling from an additional nine HAART-treated macaques infected with RT-SHIV. The HAART regimen remained the same. Larger blood draws taken regularly post-infection allowed for the longitudinal analysis of VLs using both virus load assays over the course of the study.

**Figure 3:**
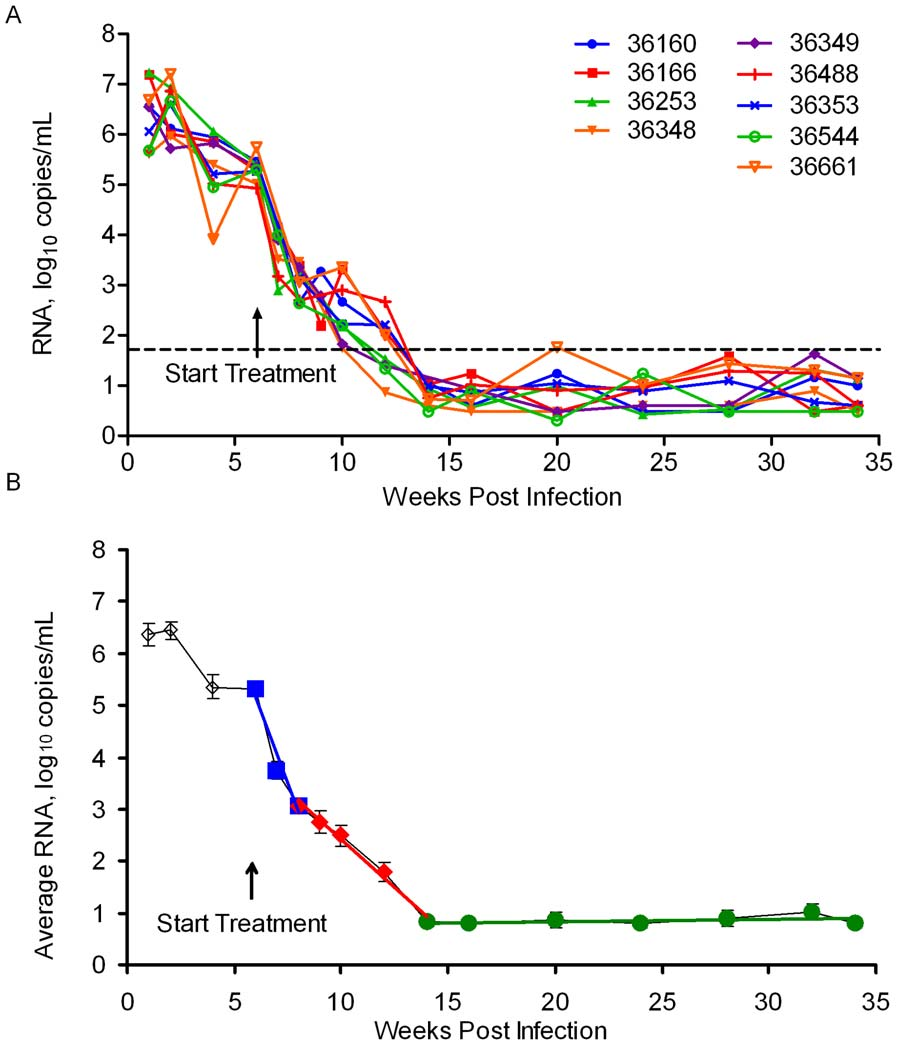
Longitudinal analysis of plasma virus loads of nine RT-SHIV-infected, HAART-treated macaques. (A) The longitudinal analysis of RT-SHIV RNA from each individual HAART-treated macaque. The dashed line indicates the limit of detection of the standard virus load assay (SVLA; 50 RNA copies/mL). (B) Linear regression analysis of the average RT-SHIV virus load for all 9 HAART-treated macaques. Error bars indicate standard error of the mean.

Plasma VLs were measured over a 34 week period using the SVLA when the VLs were more than 50 RNA copies per mL, and the UVLA for samples less than 50 RNA copies per mL (Fig. 3A). By week 12 post-infection, after six weeks of HAART, four of the nine treated macaques had a VL below the detection limit of the SVLA (Fig. 3A; dashed line). All of the treated animals were below 50 RNA copies per mL by week 14 post-infection (Fig. 3A).

Due to the small size of the macaques, most blood draws were limited to about 5 mL, resulting in plasma volumes ranging from 2–4 mL, which in turn restricted the sensitivity of the UVLA. Although most samples contained detectable levels of RNA by the UVLA, some were below the limit of detection. Based upon the plasma volume used in each assay, the theoretical limit of detection was calculated for each sample that was negative by TaqMan RT-PCR. This theoretical limit of detection was used to assign a value to the VLs, perhaps giving a slight over-estimate. Occasional 10 mL blood draws, in addition to the larger volumes of blood that were collected at necropsy, allowed for the recovery of more plasma, resulting in a more sensitive UVLA that was capable of detecting two RNA copies per mL (macaque 36544, 20 weeks post-infection).

One sample from macaque 36160 at 16 weeks post-infection showed one TaqMan replicate value of 2,020 RNA copies per mL while the other two replicates were below the limit of detection of the UVLA. The VL was also below the limit of detection in two separate SVLAs. The value of 2,020 RNA copies per mL was treated as an outlier and was excluded from the analysis. This example emphasizes the importance of performing multiple TaqMan replicates on the samples.

The average VL for all of the HAART-treated macaques over the 34 weeks of treatment was determined and linear regression analysis was performed (Fig. 3B). The best fit model of the average virus load upon initiation of HAART was characterized by three distinct phases with the following slopes: −0.37 (95% CI −0.30 to −0.43), −0.12 (95% CI −0.10 to −0.14), and 0.001 (95% CI −0.003 to 0.006; p = 0.0009). Half-lives calculated from the first two lines were: 1.8 (95% CI 1.6 to 2.3) and 5.8 (95% CI 5.0 to 6.9) days, respectively (Table 1). The third regression line (slope 0.001) indicated that the VL had reached a near steady state level, with little further decay in the average viral load over the final 20 weeks of this study.

**Figure 4:**
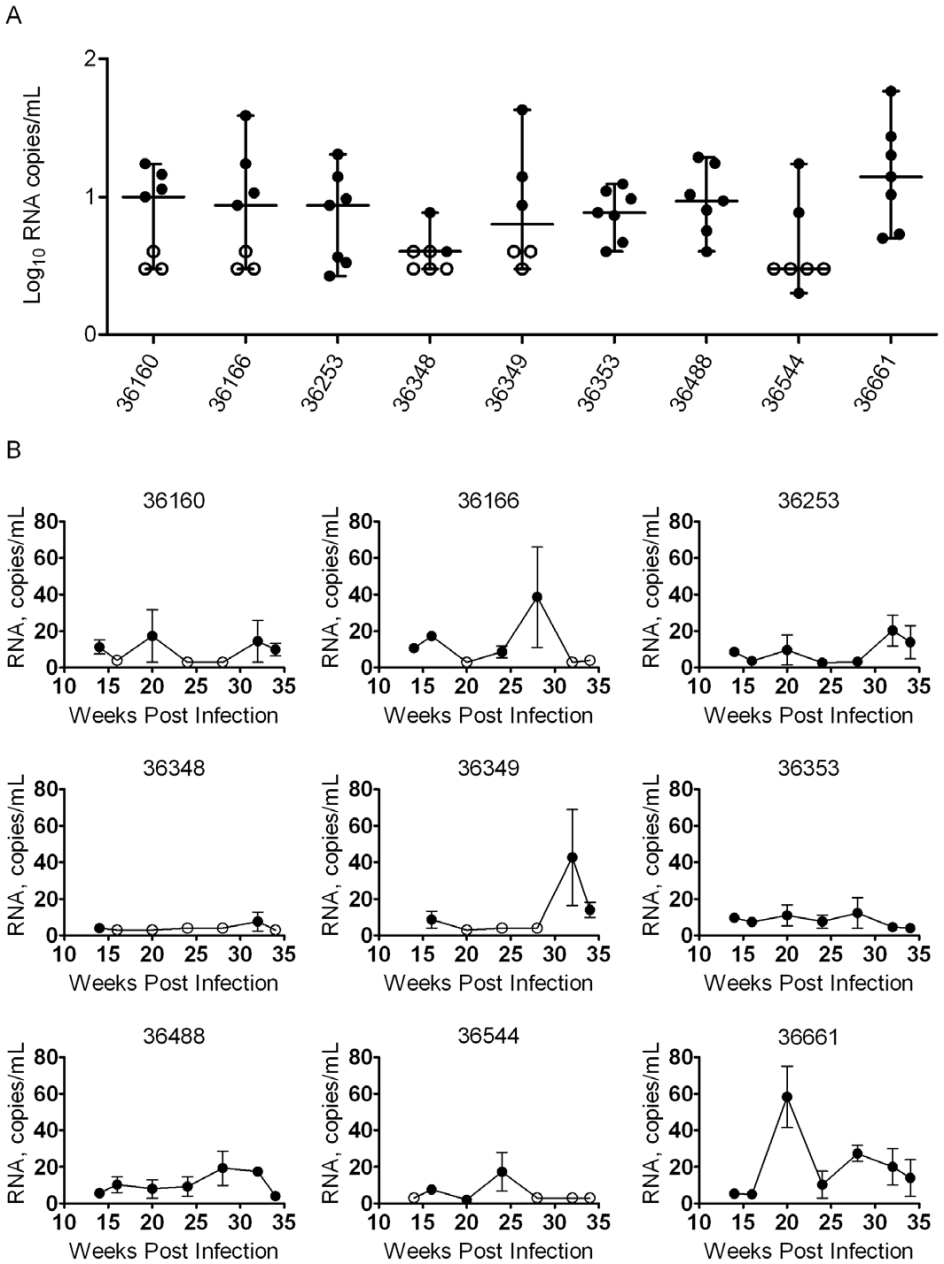
Analysis of suppressed RT-SHIV virus loads during HAART. (A) Comparison of variation in RT-SHIV plasma RNA loads during HAART from weeks 14–34 post-infection of the longitudinal study. Solid circles indicate RT-SHIV RNA copies/mL. Open circles indicate that the sample was below the level of detection of the ultracentrifugation virus load assay (UVLA). Median with the range. (B) Individual data from the nine HAART-treated macaques in the longitudinal study. Plasma samples were analyzed for levels of RT-SHIV RNA using the UVLA from 14–34 weeks post-infection. Solid circles indicate that RNA was detected in the assay. Open circles indicate that RNA levels were below the limit of detection of the UVLA. Mean plus standard error of the mean of single plasma samples analyzed by triplicate TaqMan RT-PCR. Some standard errors were too small to display on the graphs.

The VLs of each macaque from weeks 14 through 34, when viremia was suppressed below the detection limit of the SVLA, were also analyzed (Fig. 4A). A one-way ANOVA determined that the average VLs between the animals were not statistically different (*p* = 0.16). However, Bartlett's test for equal variances indicated that the standard deviations for the mean VLs of each animal were significantly different (*p*<0.0001). This result was most apparent with macaque 36348, which more consistently maintained VL suppression during HAART, with many of its plasma samples showing viral RNA levels below the detection limit of even the UVLA (Fig. 4B).

## Discussion

The results presented in this study demonstrate that low-level viremia persists in RT-SHIV-infected macaques despite treatment with a first-line HAART regimen for 28 weeks. These results further the development of the RT-SHIV/rhesus macaque model of HAART previously described by our group [37], [49]. The limit of detection of the SVLA was one of the limitations of that study; upon initiation of HAART, the VLs in the animals dropped to below the limit of detection of the assay (i.e., 50 copies of viral RNA per mL of plasma). Intermittent positive VL assays and viral rebound upon cessation of treatment suggested that viremia persists despite effective treatment, but a more sensitive virus load assay was not available.

Development of a more sensitive VL assay allowed for analysis of low-level viremia in the macaques. The data in this report demonstrated that RT-SHIV persisted at necropsy (34 weeks post-infection, 28 weeks of HAART) as well as during HAART in all of the treated animals. Their individual VLs during the live-phase, including the period of viral rebound upon cessation of HAART in several animals, has been reported recently by our group [49]. A study in pig-tailed macaques infected with another RT-SHIV (RT-SHIV_mne_) demonstrated that HAART reduced viremia in treated animals [35]. That study utilized a VL assay with a reported limit of detection of 15 RNA copies per mL [35], [46]. The UVLA that we present here is similar to the assay used in this previous study; however, our study utilized larger plasma samples, resulting in a lower limit of detection for plasma viral load. We demonstrate that low-level viremia persists below the previous limit of detection. In addition, all treated animals in our study maintained VL suppression, and no treatment failure was observed.

This is the first report of viral decay kinetics in the HAART-treated rhesus macaque model of AIDS using an RT-SHIV. These data demonstrate that upon initiation of HAART, viremia decays in a bi-phasic manner to reach a stable level that persists despite continued treatment. The viral half-lives that we estimated in the RT-SHIV-infected macaques were very similar to the values that were reported for HIV-1-infected persons on HAART (Table 1) [6], [14], [16]. However, the second phase of decay that we observed was faster than the previously reported estimates. A recent, long-term HIV-1 study that monitored virus loads for seven years found a third phase of decay with an estimated half-life of 39 weeks followed by a fourth phase with no apparent decay [6]. Our study monitored the animals through 33–34 weeks post-infection. As a result, we did not observe the third and fourth phases that were previously reported. It will be interesting to perform a long-term treatment study in RT-SHIV-infected macaques.

The macaque model allows frequent sampling and is well controlled in terms of the viral innoculum, as well as the nature of and adherence to the HAART regimen. As a result, the model might enable tracking of viral evolution during the various phases of viral decay during HAART. These viral sequences could also be compared with viral sequences obtained from tissues collected at necropsy. Monitoring changes in viral genotype during treatment could lead to an understanding of the location and extent of any residual replication during treatment.

An unpaired t test demonstrated that the average VLs from the two groups were statistically different (*p* = 0.0007): 20 RNA copies per mL in the five macaques analyzed at necropsy compared to 10 RNA copies per mL in the nine HAART-treated animals in the longitudinal study from 14–34 weeks post-infection. The animals from these two groups were both infected with RT-SHIV administered by the I.V. route, and both received the same HAART regimen initiated 6 weeks post-infection. It is possible that sampling variation contributes to some of the difference, because a single blood draw was analyzed from each of five animals at necropsy, whereas the longitudinal group involved nine animals monitored over many weeks. In a previous study, we reported that oral delivery of efavirenz had been a concern, evidenced by transient spikes in virus loads [37]. This problem was addressed by changing the food used to deliver the drug. The animal handlers did not report a delivery concern in either of the current groups. Nevertheless, we quantified drug levels by LC-MS-MS in several of the remaining plasma samples in an attempt to address the possibility that efavirenz was not being delivered adequately. Trough levels of efavirenz in the samples tested were at least 25 times the 50% effective antiviral concentration (data not shown).

The average VLs for each macaque over the final 18 weeks of HAART in the longitudinal study were not statistically different. However, Bartlett's test for equal variance indicated that the standard deviations of those VLs were different. These results demonstrate that HAART suppresses viremia to low levels in all treated macaques, but some macaques are more consistent in the maintenance of suppression than others (particularly macaques 36348 and 36353). This suggests that studies attempting to determine whether enhancing HAART with additional antiretrovirals can reduce low-level viremia should not rely strictly on average virus load as a parameter. Determining whether the enhanced treatment regimen has a detectable effect might require longitudinal studies and involve analysis such as variation around the mean and number of samples below the limit of detection. There is support for incomplete suppression of HIV-1 replication during HAART [12], [50], [51]; however, some studies have concluded that the low-level viremia is a result of the intermittent activation of latent virus [52], [53]. Resolving this issue will be critical for attempts to eradicate the virus. It is important to note that any amount of residual replication, whether it is in a drug privileged site or in major compartments of viral replication, may enable the virus to reseed reservoirs and extend the observed viral decay half-lives. Extensive analysis of virus in tissues from these macaques is ongoing in our laboratory, and might help to elucidate the extent and sites of residual replication during HAART.

As with HIV-1 in humans, residual viremia is present in RT-SHIV-infected macaques despite treatment with a first-line HAART-regimen consisting of EFV, FTC and PMPA. This nonhuman primate model will enable future studies aimed to identify and purge viral reservoirs, including enhancing HAART to achieve maximum virus load suppression in all animals as well as strategies to reactivate latent virus.
